# D-GQDs Modified Epoxy Resin Enhances the Thermal Conductivity of AlN/Epoxy Resin Thermally Conductive Composites

**DOI:** 10.3390/polym13234074

**Published:** 2021-11-24

**Authors:** Duanwei Zhang, Fusheng Liu, Sheng Wang, Mengxi Yan, Xin Hu, Mengying Xu

**Affiliations:** 1 Department of Chemistry and Materials Science, College of Science, Nanjing Forestry University, Nanjing 210037, China; zdw1023044018@163.com; 2 College of Chemical Engineering, Nanjing Tech University, Nanjing 211816, China; mengximengxiyan@163.com (M.Y.); huxin872538386@163.com (X.H.); xmy0811c@163.com (M.X.)

**Keywords:** thermal conductivity, graphene quantum dots, AlN, epoxy resin, thermally conductive composites

## Abstract

This article proposes a method of increasing thermal conductivity (λ) by improving the λ value of a matrix and reducing the interfacial thermal resistance between such matrix and its thermally conductive fillers. D-GQDs (graphene quantum dots modified by polyetheramine D400) with a π–π-conjugated system in the center of their molecules, and polyether branched chains that are rich in amino groups at their edges, are designed and synthesized. AlN/DG-ER (AlN/D-GQDs-Epoxy resin) thermally conductive composites are obtained using AlN as a thermally conductive and insulating filler, using D-GQDs-modified epoxy resin as a matrix. All of the thermal conductivity, electrically insulating and physical–mechanical properties of AlN/DG-ER are investigated in detail. The results show that D-GQDs linked to an epoxy resin by chemical bonds can increase the value of λ of the epoxy–resin matrix and reduce the interfacial thermal resistance between AlN and DG-ER (D-GQDs–epoxy resin). The prepared AlN/DG-ER is shown to be a good thermally conductive and insulating packaging material.

## 1. Introduction

Epoxy resin has been widely used as an indispensable insulating packaging material for electronic equipment and electronic components in the electronics industry because of its good electrical insulation [1,2,3]. However, the use of epoxy resin to encapsulate electronic equipment and electronic components will produces an inability to dissipate heat during operation because of lowered thermal conductivity, seriously affecting the normal operation of electronic equipment and electronic components and even leading to damage due to excessive temperature. Therefore, how the thermal conductivity of epoxy resin may be increased, in order to broaden its application range in electronic equipment and electronic-component packaging, has been attracting much more attention.

Incorporating an insulating filler of high thermal conductivity into epoxy resin to prepare thermally conductive composites is one effective means of improving thermal conductivity. The main factors affecting the thermal conductivity of composites include the intrinsic thermal conductivity of the matrix resin and fillers, the interfacial thermal resistance between the filler and the matrix resin and the integrity of the continuous thermal network structure formed by the filler, though there are many other factors. The current research is mainly focused on selecting one or more combinations of highly thermally conductive fillers to construct an improved thermal network structure, thereby improving the thermal conductivity of composites that use them.

Thermally conductive and insulating fillers used for such purpose mainly include aluminum nitride [2,4,5], graphene [6], boron nitride [7,8,9], zinc oxide [10], silicon carbide [11], magnesium oxide [12], aluminum oxide [13,14,15,16] and carbon nanomaterials [17]. When powders of thermally conductive and insulating fillers are added to an epoxy resin, the powders can form a network structure, through which heat can be transferred. Recently, AlN has been used as a thermally conductive and insulating filler to enhance the thermal conductivity of an epoxy composite. Although thermal conductivity can be increased by adding AlN to epoxy resin, it cannot achieve satisfactory results. For this reason, a variety of fillers have been added to epoxy resins to obtain a greater degree of thermal conductivity [18]. Huang et al. [2] have successfully fabricated an AlN/AgNPs/epoxy composite that exhibits better thermal conductivity, reaching 4.72 W·m^−1^·K^−1^.

The main factors affecting the construction of thermal network structures include [19,20,21,22,23] the amount of filler added, the filler’s size and morphology, its size distribution and orientation, the surface treatment of the filler particles and the interaction between the matrix and fillers. Zhu et al. [24] have investigated the effects of filler type, content, size, size distribution and morphology on the thermal conductivity of composites. Their results showed that the thermal conductivities of their composites increased with increasing filler content, and that particle size and morphology have a significant effect on thermal conductivity for BN particle-filled composites. Chen et al. [25] observed that a unique bridging between carbon nanotubes and graphene nanoplatelets led to increased contact-surface area, which can effectively promote the formation of thermally conductive networks, improving the thermal conductivity of such composites. Mostovoy and Yakovlev [26] have reported that thermally expanded graphite can affect the thermal conductivity of an epoxy composite by influencing the process of the composite’s structure formation. Hemath et al. [23] have reported that the interaction between the polymer matrix and nanofillers facilitates effective dispersion without damaging the structures of the nanofillers, which tended to optimize the thermal conductivities of treated nanocomposites. Surface treatment can significantly increase the thermal conductivity of composites by improving the filler dispersion and adhesion between the filler and the polymer matrix. Mostovoy et al. [27] have displayed that the effectiveness of h-BN surface modification, and strong chemical bonds forming at the polymer matrix/filler interface can enhance the performance of epoxy composites.

Fillers of high intrinsic thermal conductivity are beneficial for improving the thermal conductivity of composites; but, if the thermal conductivity of the filler is greater than 100 times the thermal conductivity of the matrix, the thermal conductivity of the filler is no longer a key factor affecting the thermal conductivity of the composite material. Therefore, even with the addition of a filler, the thermal conductivity of a composite only increases marginally [20]. However, there is relatively little research on reducing the thermal resistance of the matrix resin itself.

In addition, the interface thermal resistance between the filler and the matrix resin is also an important factor affecting thermal conductivity [22,28]. For epoxy composites, the particles of thermally conductive fillers are wrapped and isolated by the continuous epoxy-resin matrix. There are multiple phase interfaces between the particles of fillers and resin. The more types of thermal conductive materials that are added, the more types of phase interfaces will form in the composite. Prasher [29] has reported that the λ value of the contact zone is related to the roughness of the interface, the microscopic area of contact, the thermal conductivity of two contact substances, etc. Of particular interest here, higher thermal conductivity in both of two contact substances will reduce the interface thermal resistance between them. Due to the lower λ value of an epoxy-resin matrix, the interface thermal resistance between thermally conductive fillers and epoxy resin-matrices is higher. Therefore, thermal conductivity can be effectively increased by improving the λ value of matrix, reducing the interface’s thermal resistance between its fillers and resin. Zhang et al. [30] have reported that polymers with π–π conjugated structures can reach better thermal conductivity. Thus, the introduction of structural units such as π–π-conjugated system into cured epoxy resins is one methods of increasing its thermal conductivity.

In this work, D-GQDs with a π–π conjugated system in the center of their molecule and polyether-branched chains, rich in amino groups at their edge, was designed and synthesized. It could not only easily dissolve in the epoxy resin, but also could react with epoxy groups in the epoxy resin to form a homogeneous cured product, because D-GQDs entered the cured product by chemically bonding during the curing reaction. This indicates that there is no phase interface between D-GQDs and epoxy resin, so there is no interfacial thermal resistance between them. AlN/DG-ER thermally conductive composites were obtained using AlN as a thermal conductivity-insulating filler, using D-GQDs to modify the epoxy-resin’s matrix. The aim was to enhance the λ value of the epoxy-resin matrix, and to reduce the interfacial thermal resistance between the epoxy resin and AlN by increasing the λ value of the epoxy-resin matrix. The thermal conductivity, electrical insulation properties and physical and mechanical properties of AlN/DG-ER were investigated, in detail, to verify that the prepared AlN/DG-ER produced a good thermally conductive and insulating packaging material.

## 2. Experimental

Materials. Citric acid, aluminum nitride and ethanol of analytical grade were purchased from Aladdin Co., Ltd. (Shanghai, China). Polyetheramine (D400) was purchased from Aladdin Co., Ltd. (Shanghai, China). Epoxy resin (E-51) was purchased from Hangzhou Wuhuigang Adhesive Co., Ltd. (Hangzhou, China), and dialysis bags (MW: 500) were purchased from Viskase Company(Danbury, CT, USA).

### 2.1. Preparation of GQDs and D-GQDs

Preparation of GQDs: The GQDs materials were prepared according to [31]. In a typical procedure of GQD preparation, 30 g of citric acid is heated at 200 °C for 2 h. The product is dissolved in 20 mL ultrapure water, and then dialyzed for 72 h using a 500-Da dialysis tubing bag. The product is freeze-dried to obtain the GQDs. The equivalent value of each mole of carboxyl group in GQD molecules is 135 by titration analysis, that is, every 135 g GQDs contains 1 mole of carboxyl group.

**Scheme 1:**
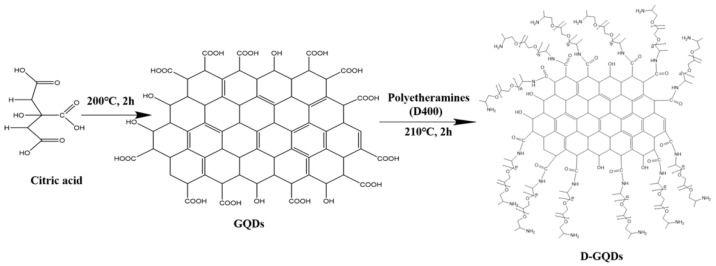
The reaction equation of D-GQDs.

Preparation of D-GQDs: The D-GQDs materials were prepared according to the amide preparation method in [32]. After dissolving GQDs into polyetheramine (D400) with a molar ratio of carboxyl groups in GQDs to polyetheramine (D400) of 1:1, the carboxyl groups in GQDs were reacted with the amino groups in the polyetheramine (D400) to form ammonium carboxylate. The obtained product was reacted at 210 °C for 2 h to induce ammonium carboxylate dehydrate to form amide. The crude product of D-GQDs was dissolved in 20 mL ultrapure water, and then dialyzed for 72 h using a 500-Da dialysis tubing bag. Finally, D-GQDs were obtained by freeze-drying. The equivalent value of each mole of amino group in the D-GQDs molecules was 517, by titration analysis. The reaction equation is shown in Scheme 1.

Characterization: The morphology of D-GQDs was observed using transmission electron microscopy (TEM) (JEM-2100 UHR). Structure characterization of D-GQDs was conducted by FT-IR spectrometer (VERTEX 80 V), in attenuated-total-reflection (ATR) mode; Raman spectrometer (Horiba Labram HR800); and X-ray photoelectron spectroscopy (XPS) (Shimadzu AXIS UltraDLD). The final FT-IR spectrum of each sample was an average of 16 scans at a resolution of 4 cm^−1^ in the wavenumber range of 400–4000 cm^−1^. The excitation wavelength of Raman spectroscopy was 780 nm. The measurement conditions of the XPS were an instrument power of 150 W, a full-spectrum scanning energy of 160 eV and narrow-spectrum scanning energy of 40 eV.

### 2.2. Sheets and Coatings Formulation and Curing

Paints: Polyetheramine (D400) was mixed with epoxy resin (E-51) to obtain ER paints. The molar ratio of the amino groups in the polyetheramine (D400) to epoxy resin (E-51) was 1:1. After D-GQDs were mixed with polyetheramine (D400), the obtained solution was mixed with epoxy resin (E-51) to obtain DG-ER paints. The molar ratio of the amino groups, both in the D-GQDs and polyetheramine (D400), to epoxy resin (E-51) was 1:1. As GQDs cannot be dissolved in epoxy resin, the GQDs were dispersed in ER paints under high shear to obtain G/ER paints. The molar ratio of the amino groups in the polyetheramine (D400) to epoxy resin was 1:1. The AlN powders were uniformly dispersed in the ER paints and DG-ER paints (the mass percentage of D-GQDs in DG-ER paint was 9 wt%) under high shear using a HR-6B laboratory homogenizer/dispersion machine (1000–35,000 rpm, Shanghai Huxi Industrial Co., Ltd., Shanghai, China) to obtain AlN/ER paints and AlN/DG-ER paints, respectively.

Tu-4 cup was used, being suitable for measuring the viscosity of paints with a viscosity of less than 150 s. After filling the Tu-4 cup with paint, the paint flowed from the standard pipe hole, and the time *t*(s) required to record it. Kinematic viscosity *v* (mm^2^·s^−1^) was obtained by

*t* = 0.223 *v* + 6.0 (23 s ≤ *t* < 150 s) (1)

Sheets: Pouring the ER paint into the mold, a 5 ± 0.5-mm thick sample of ER sheet was obtained after cured at 80 °C for 12 h. The 5 ± 0.5 mm-thick samples of the DG-ER sheet, the G/ER sheet, the AlN/DG-ER sheet and the AlN/ER sheet were obtained in the same way as that of the ER sheet, marked as x_1_-DG-ER sheet, x_2_-G/ER sheet, y_1_-AlN/DG-ER sheet and y_2_-AlN/ER sheet, where x_1_ denotes as the mass percentage of D-GQDs in DG-ER sheet, x_2_ denotes as the mass percentage of GQDs in G/ER sheet, y_1_ and y_2_ denote as the mass percentage of AlN in the AlN/DG-ER and AlN/ER sheets, respectively.

The sheets were used for thermal conductivity, surface-resistivity and volume-resistivity testing.

Coatings: For impact-strength and adhesion testing, the 20 ± 3 μm-thick layers of ER paints were coated on a tinplate substrate. The ER coating was then obtained by curing the paints at 80 °C for 12 h. The 20 ± 3 μm-thick layers of DG-ER coating, G/ER coating, AlN/DG-ER coating and AlN/ER coating were obtained in the same and marked as x-DG-ER coating, x-G/ER coating, y-Al/x-DG-ER coating and y-AlN/ER coating, respectively.

Measurement of sheets and coatings: The morphologies of sheet cross sections were characterized by scanning electron microscopy (SEM) (Hitachi, JSM-7600 F, Japan, Cu originates from the substrate not used). The thermal conductivities (λ) of the sheets were measured by the thermal conductivity tester (TPS2500, Hot Disk, Sweden) with transient plate heat-source method accoriding to the reference standard ISO22007-2. The surface (*ρ*_s_) and volume resistivities (*ρ*_v_) of the sheets were measured by an ultra-high-resistance micro current meter (ZC-36, Shanghai Precision Scientific Instrument Co., Ltd. (Shanghai, China) at 20 °C and 60% relative humidity. The contact angles of the sheets were measured using an optical contact-angle tester (DSA100, KRUSS Company, Hamburg, Germany) at 20 °C, 60% relative humidity and 15 μL of dropped water. The decomposition behavior of sheets was measured using a TGA instrument (TG 209 F1, NETZSCH, Germany) from 30 to 800 °C at 20 °C min^−1^ in a nitrogen atmosphere. The adhesion of the coatings was measured with the cross-cut test and classified by ISO2409 standards. The impact strength of the coatings was tested by a paint film impactor (QCJ-100, Shengda Measuring Instrument Tool Factory, Jinghai County, Tianjin, China).

## 3. Results and Discussion

### 3.1. Characterization of D-GQDs

#### 3.1.1. Morphology Characterization

**Figure 1:**
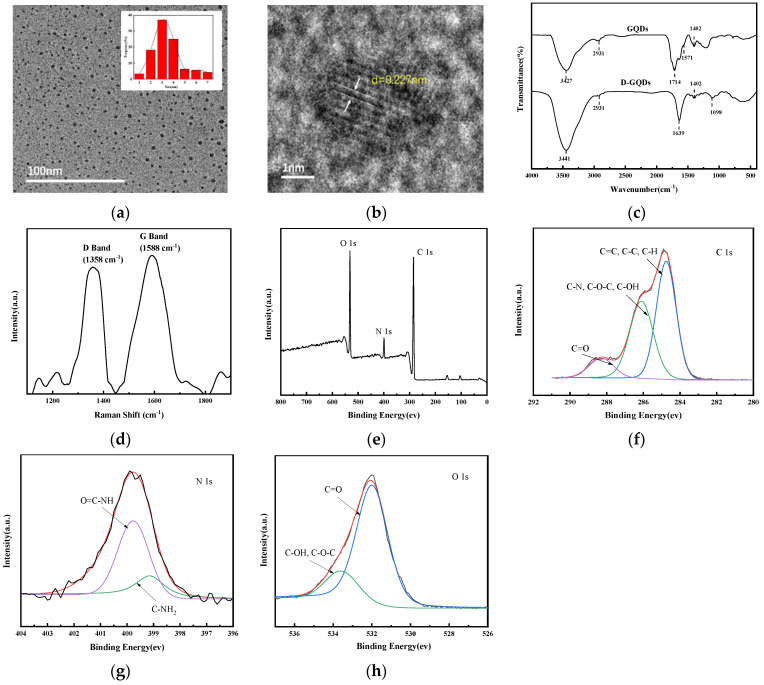
(**a**) TEM image (inset: particle−size distribution), (**b**) HRTEM image, (**c**) FTIR spectra, (**d**) Raman spectra, (**e**) XPS spectra and high−resolution XPS spectra of (**f**) C 1 s, (**g**) N 1 s, (**h**) O 1 s of D-GQDs.

From Figure 1a, the picture reveals that the D-GQDs particles exhibit a nearly spherical shape and good dispersibility. The statistic result from TEM image indicated that the particle sizes were 2–7 nm, as shown in the inset of Figure 1a. From the HRTEM image in Figure 1b, clear lattice fringes demonstrate the well-crystalline structure, and the autocorrelated HRTEM lattice image shows a 0.227-nm lattice fringe assigned to the (1120) lattice fringes of graphene [33,34].

#### 3.1.2. Structure Characterization

Figure 1c exhibits the FT-IR spectra of GQDs and D-GQDs. The FT-IR spectrum of GQDs shows that the absorption peak at 3000–3600 cm^−1^ implies the presence of –OH. The absorption peaks at 2931 cm^−1^ and 1571 cm^−1^ belong to the stretching vibration of =C–H and C=C in aromatic rings [35], respectively. This can indirectly indicate the presence of graphene structure. The peaks at 1714 cm^−1^ and 1402 cm^−1^ belong to the stretching vibrations of C=O in a carboxylic group and phenolic hydroxyl, O–H [36,37,38], respectively. This shows that there were COOH and OH groups in the GQDs. The FT-IR spectrum of the D-GQDs shows that the absorption peak observed at 3000–3600 cm^−1^ relates to –OH, and the N–H tensile absorption peak was also in this range [36]. The absorption peak at 2931 cm^−1^ also belongs to the stretching vibration of =C–H in aromatic rings [35]. The absorption peak at 1571 cm^−1^, attributed to C=C in aromatic rings, overlapped with the absorption peak at 1639 cm^−1^. The absorption peaks at 1402 cm^−1^ and 1098 cm^−1^ are attributed to phenolic-hydroxyl O–H [37] and C–O–C stretching vibrations in polyetheramine branches [35], respectively. In contrast with the FT-IR spectrum of the GQDs, the absorption peak at 1714 cm^−1^ disappeared, meaning the COOH group also disappeared, and the absorption peak at 1639 cm^−1^ appears attributed to C=O in secondary amides [36]. This indicates that the –COOH at the edge of the GQDs reacted with the –NH_2_ in D400 to form amides. Therefore, the FTIR results show the obtained products were GQDs and D-GQDs, respectively.

The structural information of the D-GQDs’ quantum graphene dots was examined by Raman spectroscopy, as shown in Figure 1d. The sample displayed two peaks at 1358 cm^−1^ and 1588 cm^−1^, named the D and G bands, respectively. As shown in Figure 1d, the ratio of the intensities (I_D_/I_G_) of these characteristic bands was 0.91, which suggests the D-GQDs’ crystalline nature and integrated graphite structure [37,39].

From the XPS spectra in Figure 1e, the characteristic spectral lines for N, C and O can be seen. From Figure 1f, the C 1 s spectrum can be divided into three peaks. The peak at 284.9 eV is ascribed to C=C, C–C and C–H bonds [40]. The peak at 286.1 eV is assigned to C–N, C–O–C and C–OH bonds [3]. The peak at 288.3 eV is related to C=O bonds [40]. From Figure 1g, the N 1 s spectra can be divided into two peaks, at 399.8 eV and 399.2 eV, which are ascribed to O=C–NH and C–NH_2_ bonds [40]^,^ respectively. From Figure 1h, the O 1 s spectrum can be divided into two peaks, centered on 532.0 eV and 533.6 eV, which are related to the C–OH, C=O and C–O–C bonds, respectively [40]. All these further confirm that C=O, C=C, O=C–NH, C–OH, C–NH_2_ and C–O–C are formed in the structure of D-GQDs, which fits with the FTIR results.

### 3.2. DG-ER Sheet

#### 3.2.1. Surface Morphology

**Figure 2:**
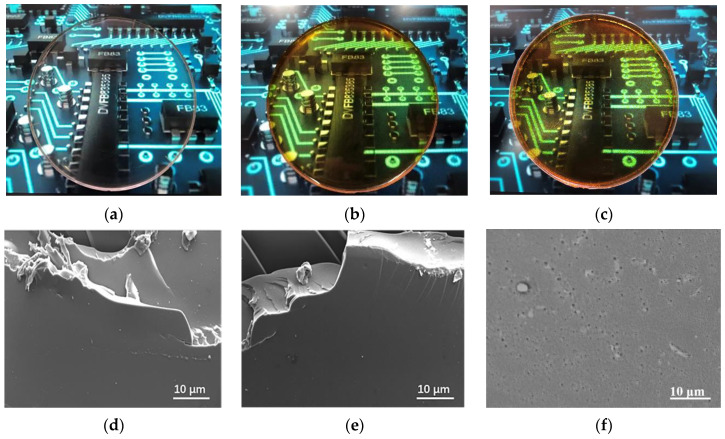
Photographs of (**a**)ER sheet, (**b**) 9-DG-ER sheet and (**c**) 9-G/ER sheet; SEM images of the cross section of (**d**) ER sheet, (**e**) 9-DG-ER sheet and (**f**) 9-G/ER sheet.

Figure 2a–c shows the photographs of the ER sheet, 9-DG-ER sheet and 9-G/ER sheet. Figure 2a shows that the ER sheet is colorless and transparent. In Figure 2b, the 9-DG-ER sheet is shown to be light yellow and transparent, as D-GQDs can be dissolved in epoxy resin paints. In Figure 2c, we see that the 9-G-ER sheet is also light yellow and transparent, but some black, incompatible GQDs particles are evenly distributed in the 9-G/ER sheet.

SEM analyses of the cross sections of the ER, 9-DG-ER and 9-G/ER sheets are presented in Figure 2d–f. As shown in Figure 2d, the cross-sectional view of the ER sheet is smooth and homogeneous. It can be observed from Figure 2e that the cross section of 9-DG-ER sheet is also smooth and homogeneous. This reveals the reason for D-GQDs’ good compatibility with epoxy resins; the amino groups in D-GQDs can react with epoxy groups in epoxy resins to promote D-GQDs’ entry into the cured product by chemical bonding. As shown in Figure 1a, the D-GQDs particle sizes are 2–7 nm. Thus, the nano-graphene structure is chemically bonded to the cured product without altering the smoothness or uniformity of the ER sheet, and while preserving a uniform phase. Compared with the D-GQDs, GQDs particles are incompatible with epoxy resin; as seen in Figure 2f, there are particles on the cross section of the ER sheet, meaning the GQDs’ particles are dispersed within the ER sheet.

#### 3.2.2. Thermal Conductivity of the DG-ER Sheet

**Figure 3:**
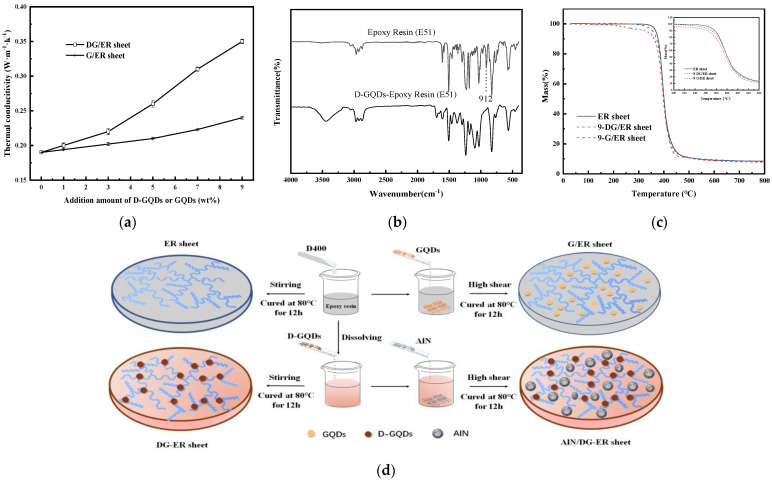
(**a**) The thermal conductivity of the DG-ER and G/ER sheets; (**b**) the FT−IR spectra of epoxy−resin (E-51) and D-GQDs–epoxy–resin (E-51) sheet; (**c**) the TGA curves of ER sheet, 9-DG-ER sheet and 9-G/ER sheet (inset: an enlarged view of the curves); (**d**) schematic representations of the conductive mechanism.

Figure 3a shows the relation between the added amount of D-GQDs and GQDs and the thermal conductivity (λ) of resulting ER sheet. When no D-GQDs and GQDs were added, the value of λ was 0.19 W·m^−1^·K^−1^. The values of λ of both the DG-ER sheet and the G/ER sheet increased with increasing amounts of D-GQDs and GQDs. This demonstrates the reason for both D-GQDs and GQDs molecules having rigid structures in their π–π conjugated systems in their central regions, as shown in Scheme 1. As the π–π conjugated system is important for obtaining high thermal conductivity [30,41], the λ values increase with the increasing D-GQDs and GQDs in the DG-ER sheet and G/ER sheets, respectively. Yet, λ values for the DG-ER sheet were larger than those of the G/ER sheet at the same added contributions. As shown in Scheme 1, the D-GQDs molecules contain lots of polyether branched chains with amino groups at their edges, such that they can be dissolved in epoxy resin. In the preparation process of the DG-ER sheet, after replacing a portion of the polyetheramine (D400) with D-GQDs, D-GQDs and D400 were mixed with epoxy resin. The amino groups in the D-GQDs and D400 react with the epoxy groups of epoxy resin and undergo cross-linking and curing reactions, promoting D-GQDs’ entry into the cured product through chemical bonding, obtaining the transparent DG-ER sheet shown in Figure 2b. In order to prove the chemical interaction of the D-GQDs with the epoxy resin, the D-GQDs were mixed with epoxy resin (E-51) to obtain the D-GQDs–epoxy resin (E-51) paint. The molar ratio of the amino groups in the D-GQDs to epoxy resin (E-51) was 1:1. Pouring the D-GQDs–epoxy-resin (E-51) paint into the mold, a 5 ± 0.5-mm thick sample of the D-GQDs–epoxy-resin (E-51) sheet was obtained after being cured at 80 °C for 12 h. Figure 3b shows the FT-IR spectra of the epoxy-resin (E-51) and D-GQDs–epoxy-resin (E-51) sheet. From Figure 3b, observing the FT-IR spectra of the epoxy resin (E-51), the absorption peak at 912 cm^−1^, attributed to epoxy rings, disappears at the FT-IR spectra of D-GQDs-epoxy-resin (E-51) sheet, meaning that the amino groups in the D-GQDs reacted with the epoxy groups in the epoxy resin. This indirectly indicates that D-GQDs enter the cured product through chemical bonding. The structure of the chemical bonds linking the D-GQDs and the epoxy resin illustrates that there is no interface between the D-GQDs and the epoxy resin, which means that no interfacial thermal resistance exists between the D-GQDs and epoxy resin in the DG-ER sheet, as shown in Figure 3d. Compared with the D-GQDs molecules, the GQDs molecules are incompatible with epoxy resin and are dispersed within the ER sheet, as shown in Figure 2c,f. Therefore, there is interfacial thermal resistance between the GQDs and the epoxy resin, as shown in Figure 3d, which hinders heat transfer. Thus, the values of λ for the DG-ER sheet are larger than those of the G/ER sheet at the added contributions. When the added amount of D-GQDs was 9 wt%, the value of λ reached 0.35 W·m^−1^·K^−1^. Further increasing the added amounts of D-GQDs results in the D-GQDs inability to be completely dissolved in the epoxy resin.

The TGA curves of the ER, 9-G/ER and 9-DG-ER sheets are shown in Figure 3c. Figure 3c shows that the onset decomposition temperature of the ER sheet, 9-G/ER sheet and 9-DG-ER sheet is 374.5, 371 and 366.5 °C, respectively. When temperatures exceeded 450 °C, three samples basically decomposed, leaving a residual amount of about 10%. It can be seen that the TGA curves of the three samples basically overlap, but the onsets of their decomposition temperatures are slightly different.

#### 3.2.3. Electrical Insulation Properties of DG-ER Sheet

**Figure 4:**
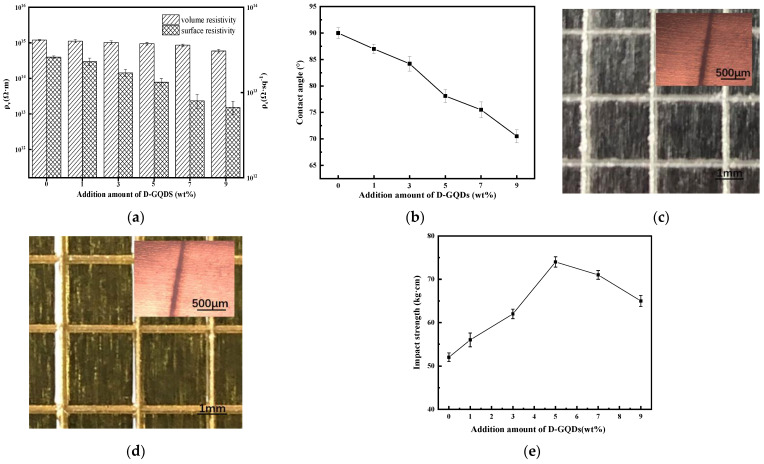
(**a**) The surface resistivity and volume resistivity and (**b**) contact angles of the DG-ER sheet. Also shown are photographs of the surface of the notches in the (**c**) ER coating (inset: SEM image) and (**d**) DG-ER coating (inset: SEM image), and (**e**) The impact strength of DG-ER coating.

For engineering applications of these thermally conductive composites, electrical insulation properties, such as surface resistivity (*ρ*_s_) and volume resistivity (*ρ*_v_), are very important. Figure 4a demonstrates the values of *ρ*_s_ and *ρ*_v_ of the DG-ER sheet. From Figure 4a, we see that with increasing amounts of D-GQDs, the surface resistivity and volume resistivity decrease, meaning the electrical insulating property of the sheet decreased. As shown in Scheme 1, the molecular formula of the D-GQDs contains many polar hygroscopic groups, such as hydroxyl, amide and amino groups, ether bonds, etc., which increases the sheet’s polarity and hygroscopic properties, reducing the values of *ρ*_s_ and *ρ*_v_.

These changes of polarity and hygroscopic properties can be confirmed by water-contact angles. Figure 4b displays the decreasing contact angles of the DG-ER sheets with increasing amounts of added D-GQDs, indicating the increase of the polarity and hygroscopic properties of the sheet. This provides evidence for the change of the values of *ρ*_s_ and *ρ*_v_ discussed above. When the added amount of D-GQDs is 9 wt%, the corresponding surface resistivity and volume resistivity of the DG-ER sheets reached 6.7 × 10^12^ Ω·sq^−1^ and 5.9 × 10^14^ Ω·m, respectively, meaning that the sheets still had good electrical insulation properties.

#### 3.2.4. Physical and Mechanical Properties of DG-ER Sheet

Physical and mechanical properties, such as adhesion and impact strength, are also very important for electronic equipment and electronic component packaging. According to the photographs of the surface of the notches in the ER and DG-ER coating (inset: SEM image) shown in Figure 4c,d, the results of the adhesion tests show that the edges of the incisions of the ER and DG-ER coatings are smooth and the edges of the lattice are free of flaking. So, the adhesion classification of both coatings is zero, according to the ISO-2490 standard [42], indicating a non-obvious change in adhesion after the introduction of the D-GQDs.

The results of the impact-strength testing of the DG-ER coating are shown in Figure 4e. The impact strength of the ER sheet was 52 kg·cm. With increasing amount of added D-GQDs, the impact strength of the DG-ER coating first increased and then decreased. It can be seen from Scheme 1 that D-GQD molecules contain multiple amino groups, which results in greater functionality. When the amount of added D-GQDs was small, the crosslink density of the cured product increased with increasing amount of added D-GQDs. Thus, the cohesive strength also increased, resulting in increased impact strength. When the amount of added D-GQDs exceeded 5 wt%, the crosslink density of the cured product was too high, introducing increased brittleness to the cured product, thus decreasing its impact strength. The impact strength of the DG-ER coating was 65 kg·cm when the amount of added D-GQDs was 9 wt%, higher than that of the ER coating. This indicates that both the thermal conductivity and impact strength of the ER coating were improved by adding D-GQDs.

### 3.3. Thermal Conductivity of AlN/DG-ER Sheet

#### 3.3.1. Surface Morphology

**Figure 5:**
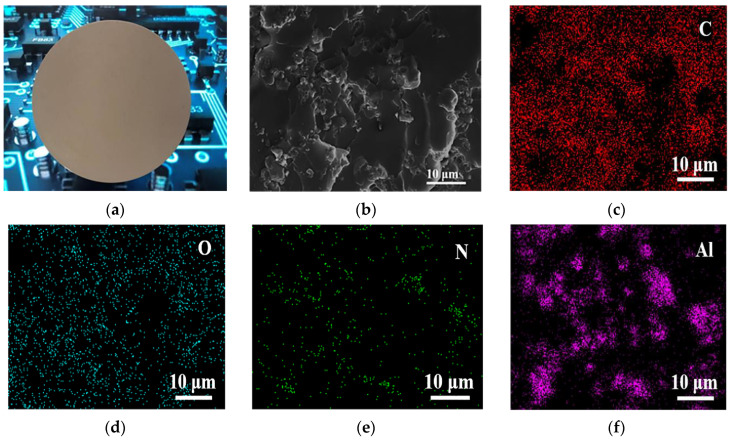
(**a**) Photographs and (**b**) SEM images of 20-AlN/DG-ER sheet, EDS of (**c**) C, (**d**) O, (**e**) N and (**f**) Al.

A photograph of the 20-AlN/DG-ER sheet is presented in Figure 5a. Compared with the photograph of the 9-DG-ER sheet, shown in Figure 2b, it can be seen that the 20-AlN/DG-ER sheet became opaque after adding the AlN particles.

The SEM analysis of the cross section of the 20-AlN/DG-ER sheet is shown in Figure 5b. Figure 5b also shows that the AIN particles are uniformly distributed between the 9-DG-ER-matrix resin; the interconnection of its AIN particles was advantageous for the formation of thermally conductive pathways in the 9-DG-ER-matrix resin. Figure 5c–f depcits the EDS analyses of the 20-AlN/DG-ER sheet, while Figure 5c–f displays that the elements C, O, N and Al are evenly distributed inside the sheet, illustrating the uniform distribution of AlN nanoparticles inside the sheet. It can be seen from the distribution map of Al that the AlN particles primarily formed a continuous network morphology inside the 9-DG-ER sheet, though some AlN particles aggregated together. High thermal conductivity can be achieved when producing this continuous network.

#### 3.3.2. Thermal Conductivity of AlN/DG-ER Sheet

**Figure 6:**
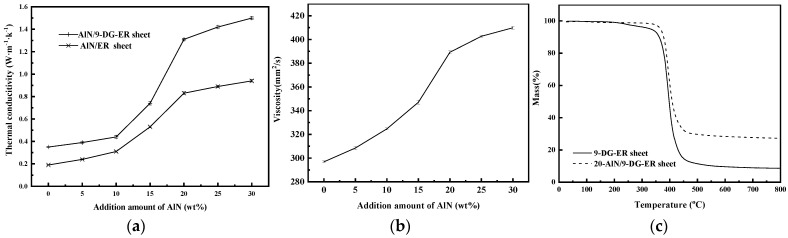
(**a**) The thermal conductivity of the AlN/DG-ER and AlN/ER sheets; (**b**) the viscosity curve of the AlN/DG-ER paint and (**c**) the TGA curves of the 20-AlN/DG-ER and 9-DG-ER sheets.

Using AlN powders as a thermally conductive and insulating filler can enhance the thermal conductivity of epoxy composites by forming a continuous thermally conductive network structure within them. Jiao et al. [43] have achieved λ values in an epoxy composite of 0.42 W·m^−1^·K^−1^ by adding 17.6 vol% of AlN. Chung and Lin [44] have reported that, after adding 20 vol% of AlN to their epoxy composite, the λ value of the composite was about 0.6 W·m^−1^·K^−1^. Figure 6a presents a comparison of thermal conductivities for the AlN/DG-ER sheet and the AlN/ER sheet at the same amounts of added AlN addition. The λ values of both the AlN/DG-ER sheet and AlN/ER sheet were improved with increasing amounts of AlN, because the AlN powders were evenly dispersed in the AlN/DG-ER and AlN/ER sheets, forming a continuous thermally conductive network structure to transfer heat, as shown in Figure 3d.

When the amount of added AlN was less than 10 wt%, the AlN particles were dispersed in the ER and 9-DG-ER sheets and isolated from each other; the AlN particles could not form a continuous thermally conductive network structure. Heat transfer, here, mainly relies on the matrix materials, ER and 9-DG-ER, respectively. So, their thermal conductivities were lower, while the λ value of the AlN/DG-ER sheet was higher than that of the AlN/ER sheet, due to the higher λ value of 9-DG-ER. Comparing the two thermal conductivity curves, they are basically parallel, indicating that their increases in thermal conductivity were alike. This is due to small amounts of added AlN, which caused the phase interface area between AlN and the matrix ER or 9-DG-ER to be small, meaning the interfacial thermal resistance has little effect on thermal conductivity, further confirming that the heat is mainly transferred by ER and 9-DG-ER, respectively. When the amount of added AlN was between 10–20 wt%, the sheets’ thermal conductivities increased rapidly, because AlN particles are evenly dispersed in the matrix ER and 9-DG-ER, respectively, forming a continuous thermally conductive network structure. When the amount of added AlN was 20 wt%, the λ values of the AlN/DG-ER sheet and the AlN/ER sheet reached 1.31 and 0.83 W·m^−1^·K^−1^, respectively, and the λ value of the AlN/DG-ER sheet was higher than previously reported [43,44]. When the amount of AlN was further increased, the λ values of both the AlN/DG-ER sheet and the AlN/ER sheet increased more slowly. This indicates that the formed thermally conductive network is almost complete at an AlN addition of 20 wt%. Comparing the two thermal conductivity curves, the increasing in thermal conductivity of the AlN/DG-ER sheet is higher than that of AlN/ER sheet when the addition amount of AlN is between 10–20 wt%. This is due to the increasing phase interface area between AlN and the matrix ER or DG-ER, resulting in the influence of the interfacial thermal resistance on thermal conductivity to increase with increasing amounts of added AlN; higher thermal conductivity between the two contact substances will reduce the interfacial thermal resistance between them [30]. The interfacial thermal resistance between AlN and ER is larger than that between AlN and 9-DG-ER because of the higher thermal conductivity of 9-DG-ER. Therefore, the increasing λ value of the AlN/DG-ER sheet is larger than that of the AlN/ER sheet. When the addition amount of AlN was more than 20 wt%, although the λ values increased slowly, the increased thermal conductivity of the AlN/DG-ER sheet is still higher than that of the AlN/ER sheet, which can also be explained by the influence of interfacial thermal resistance. With increasing AlN content, the phase-interface area also increases and the contribution of interfacing thermal resistance in the total thermal resistance increases as well. Thus, the degree of improvement in thermal conductivity in the AlN/DG-ER sheet was higher than that of the AlN/ER sheet because of its lower interface thermal resistance.

The viscosity curve of the AlN/DG-ER paint, shown in Figure 6b, displays that there was an increase in the viscosity of the AlN/DG-ER paint due to the addition of AlN. Yet, the change in the viscosity of the epoxy composition with the introduction of 0–30 wt% of AlN had little effect on the preparation or use of these composites.

Figure 6c presents the TGA curves of the 20-AlN/DG-ER and 9-DG-ER sheets. The onset decomposition temperature of the 20-AlN/DG-ER sheet was 376.9 °C, which was higher than that of 9-DG-ER. This indicates that there is a slight improvement in the thermal stability by the addition of AlN. When the temperature exceeded 450 °C, approximately 80% of the 20-AlN/DG-ER sheet decomposed, because the component AlN cannot decompose in temperatures of 450–800 °C. All of these explain the 20-AlN/DG-ER sheet’s good thermal stability.

#### 3.3.3. Electrical Insulation Properties of the AlN/DG-ER Sheet

**Figure 7:**
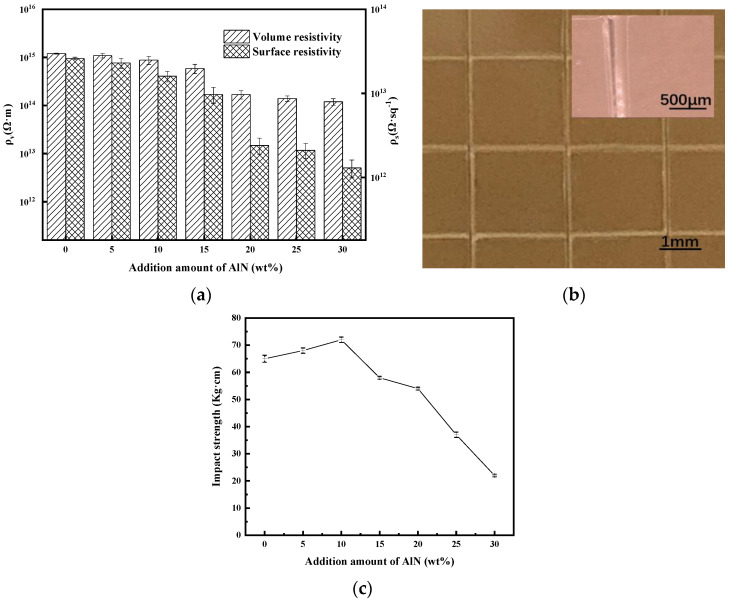
(**a**)The surface resistivity and volume resistivity of the AlN/DG-ER sheet, (**b**) a photograph of the surface of the notches in the AlN/DG-ER coating (inset: SEM image) and (**c**) the impact strength of the AlN/DG-ER coating.

Figure 7a shows the changes of *ρ*_s_ and *ρ*_v_ of the AlN/DG-ER sheet with additional amounts of AlN. From Figure 7a, we see that surface resistivity and volume resistivity decrease with increasing AlN, since the values of both *ρ*_s_ and *ρ*_v_ of AlN are lower than those of epoxy resins. When the amount of added AlN was 30 wt%, the corresponding values of *ρ*_s_ and *ρ*_v_ of AlN/DG-ER sheet reached 1.3 × 10^12^ Ω·sq^−1^ and 1.2 × 10^14^ Ω·m, respectively, which means that the sheet preserved its electrically insulating properties.

#### 3.3.4. Physical and Mechanical Properties AlN/DG-ER Sheet

Figure 7b shows a photograph of the surface of the notches in the AlN/DG-ER coating (inset: SEM image). From Figure 7b, it can been observed the coating did not detach; the adhesion classification of the AlN/DG-ER coatings is zero, indicating a non-obvious change in adhesion after the introduction of D-GQDs and AlN.

Figure 7c presents the testing results of the impact strength of the AlN/DG-ER coatings. When the amount of adde AlN was less than 10 wt%, the rigid AlN particles dispersed uniformly within the AlN/DG-ER coatings. It is beneficial to transfer stress and reduce the impact energy in a stress-transfer process, as this increases impact strength—here, the of AlN/DG-ER coatings. So, the impact strengths of the AlN/DG-ER coatings increased with increasing amounts of AlN. When the amount of added AlN exceeded 10 wt%, due to the nature of the particles, some of the AlN particles will agglomerate (as shown in Figure 5f) and form many micropores inside the agglomerated particles. When the coating is impacted by an external force, the cracks generated inside the coating will expand to become macro-cracks due to these micropores in the coating, reducing its impact resistance. Therefore, when the amount of added AlN exceeded 10 wt%, the impact strength of the coating rapidly began decreasing with further increasing AlN. The impact strength of the AlN/DG-ER coating was 54 kg·cm when the amount of added AlN was 20 wt%, which is still close to the value of the ER coating, 52 kg·cm. When the AlN content was 30 wt%, impact strength fell to only 22 kg·cm, which was inadequate to the impact-strength requirements of the coating. Figure 6a shows that when the amount of added AlN exceeded 20 wt%, the hitherto-increased λ values of the AlN/DG-ER sheet began decreasing, suggesting that the ideal AlN content is 20 wt%.

## 4. Conclusions

In summary, D-GQDs with a π–π conjugated system in the center of their molecules, and with polyether branched chains, rich in amino groups, at their edges were designed and synthesized. AlN/DG-ER thermally conductive composites were obtained using AlN as a thermally conductive and insulating filler and D-GQDs-modified epoxy resin as a matrix. The thermal conductivity, electrically insulating properties and the physico-mechanical properties of AlN/DG-ER were investigated in detail. The results showed that the thermal conductivities of AlN/DG-ER sheets can be effectively increased due to the increase in thermal conductivity contributed by 9-DG-ER and a reduction in the interfacial thermal resistance between the 9-DG-ER and AlN. When the amount of added AlN was only 20 wt%, the λ values of the AlN/DG-ER sheets reach 1.31 W·m^−1^·K^−1^, 6.89 times that of the ER matrix, while preserving the sheets’ electrically insulating properties. Both the adhesion and the impact strength of the AlN/DG-ER coating were close to those of the ER coating when the AlN content was 20 wt%. D-GQDs preparations, which were shown to increase the λ values of the matrix and reduce interfacial thermal resistance, are an effective method of preparing a good thermally conductive and insulating packaging material.

## Data Availability

Not applicable.
